# Jarin-1, an inhibitor of JA-Ile biosynthesis in *Arabidopsis thaliana*, acts differently in other plant species

**DOI:** 10.1080/15592324.2023.2273515

**Published:** 2023-10-30

**Authors:** Ming Zeng, Franziska Krajinski, Nicole M. van Dam, Bettina Hause

**Affiliations:** a Molecular Interaction Ecology, German Centre for Integrative Biodiversity Research (iDiv) Halle-Jena-Leipzig, Leipzig, Germany; b Institute of Biodiversity, Friedrich Schiller University Jena, Jena, Germany; c General and Applied Botany, Institute of Biology, Universität Leipzig, Leipzig, Germany; d Plant Biotic interactions, Leibniz Institute of Vegetable and Ornamental Crops (IGZ), Großbeeren, Germany; e Leibniz Institute of Plant Biochemistry, Department of Cell and Metabolic Biology, Halle, Germany

**Keywords:** Jarin-1, root length, jasmonic acid methyl ester (MeJA), jasmonic acid isoleucine (JA-Ile)

## Abstract

Jasmonates (JAs), including jasmonic acid (JA) and its biologically active derivative JA-Ile, are lipid-derived plant signaling molecules. They govern plant responses to stresses, such as wounding and insect herbivory. Wounding elicits a rapid increase of JA and JA-Ile levels as well as the expression of JAR1, coding for the enzyme involved in JA-Ile biosynthesis. Endogenous increase and application of JAs, such as MeJA, a JA methylester, result in increased defense levels, often accompanied by diminished growth. A JA-Ile biosynthesis inhibitor, jarin-1, was shown to exclusively inhibit the JA-conjugating enzyme JAR1 in *Arabidopsis thaliana*. To investigate whether jarin-1 does function similarly in other plants, we tested this in *Medicago truncatula*, *Solanum lycopersicum*, and *Brassica nigra* seedlings in a root growth inhibition assay. Application of jarin-1 alleviated the inhibition of root growth after MeJA application in *M. truncatula* seedlings, proving that jarin-1 is biologically active in *M. truncatula*. Jarin-1 did not show, however, a similar effect in *S*. *lycopersicum* and *B. nigra* seedlings treated with MeJA. Even JA-Ile levels were not affected by application of jarin-1 in wounded leaf disks from *S. lycopersicum*. Based on these results, we conclude that the effect of jarin-1 is highly species-specific. Researchers intending to use jarin-1 for studying the function of JAR1 or JA-Ile in their model plants, must test its functionality before use.

The signaling hormone jasmonic acid (JA) regulates up to 85% of wound- and herbivore-induced genes in *Arabidopsis thaliana* leaves.^1^ Besides regulating the response to herbivore attacks, JAs are also involved in regulating plant growth processes, such as primary root growth, reproductive development, and leaf senescence.^2,3^ The amino acid conjugate JA-Ile has been identified as the main endogenous bioactive JA molecule.^4^ However, recent studies report other endogenous bioactive JA molecules, such as JA conjugated with other amino acids or hydroxylated JA-Ile.^5^

The JA signaling pathway can integrate primary and specialized metabolism *via* the regulation of repressor-transcription factor complexes, which may be associated with re-allocation of carbon and recalibration of growth rates.^6^ Therefore, the activation of inducible plant defenses often results in reduced growth and development.^7^ This is commonly referred to as the growth-defense trade-off.^8^ The mechanisms of growth-defense trade-offs have been widely studied in plants against insect herbivory (reviewed in).^8,9^ Even knowledge about how to uncouple this mechanism has still been less explored, researchers are always searching for an effective and efficient approaches to solve this scientific question.

A previously described JA-Ile biosynthesis inhibitor, jarin-1, was firstly shown to exclusively inhibit the JA-conjugating enzyme JAR1 in *A. thaliana* and *Cardamine hirsuta*. Chemically, jarin-1 is a derivative of the alkaloid cytisine and harbors a 3-aminocytisine residue linked to two different carboxylic acid residues.^10^ Jarin-1 application prevented active JA signaling by blocking the formation of JA-Ile, the bioactive form of JA.^10,11^ Thereafter, jarin-1 has been used to inhibit JA induced responses in other species, such as strawberry, tomato and potato.^12–14^ However, the effect of jarin-1 in inhibiting JA-Ile biosynthesis was not always evidenced as done for *A. thaliana*.^10^ This leads to the hypothesis that the function of this chemical inhibitor is highly dependent on the plant species used.

In this study, we investigated whether jarin-1 does act on relieving root growth inhibition by jasmonates in other plant species, such as *Medicago truncatula*, *Solanum lycopersicum* and *Brassica nigra*. We performed the well-established root growth inhibition assay by treatment of seedlings from *M. truncatula*, *S. lycopersicum* and *B. nigra* with MeJA and applied jarin-1 simultaneously with MeJA to check, whether jarin-1 prevents the root growth inhibition or not. Our results show clearly that jarin-1 did not rescue the inhibitory effect of MeJA in all plant species tested.

First, to test whether jarin-1 counteracts MeJA function in *M. truncatula*, we used seedlings grown *in vitro* and treated them with different concentrations of jarin-1 (0, 5, 10 or 30 µM). Half of the plants were simultaneously treated with 10 µM MeJA, a treatment leading to significantly decreased root growth in various plant species.^15^ Root length was determined eight days after treatment (Figure 1). Treatment with 30 µM jarin-1 alone had a clear negative effect on root growth, which was also observed on seedlings treated with 10 µM jarin-1, although to a smaller extent (Figure 1). Simultaneous application of 5 or 10 µM jarin-1 partially alleviated the root growth inhibition by MeJA. This demonstrated that, like in *A. thaliana*,^10^ jarin-1 can partially mitigate MeJA-induced root growth inhibition in *M. truncatula*. Based on our results and the results from the previous report in *A. thaliana*, we selected the 10 µM jarin-1 treatment to test its effect on MeJA-root growth in seedlings of *S. lycopersicum* and *B. nigra*. Application of 10 µM jarin-1 had, however, no effect on the MeJA-induced root growth inhibition in seedlings of *S. lycopersicum* and *B. nigra*. In other words, seedlings treated with 10 µM MeJA and 10 µM jarin-1 simultaneously, had similar root lengths as seedlings treated with 10 µM MeJA alone (Figure 2, Table 1). This suggests that the function of jarin-1 might dependent on the plant species. To check the effect of jarin-1 on the endogenous rise of JA-Ile in tomato leaves upon wounding, leaf disks were treated with 30 µM jarin-1 for one hour followed by wounding with forceps. This concentration was selected since 10 µM jarin-1 did not rescue MeJA-induced root growth inhibition of *S. lycopersicum* seedlings and 30 µM jarin-1 was most effective in inhibiting JA-Ile production in *A. thaliana* plants.^10^ Determination of JA-Ile levels at one hour after wounding revealed, however, that jarin-1 had no effect on the wound-induced biosynthesis of JA-Ile in tomato leaf disks (Figure 3). Based on our results, we propose that the function of jarin-1 as inhibitor of formation of active jasmonates depends on the plant species. To use it, researchers should test the activity of jarin-1 in their model plants before addressing specific scientific questions related to the function of JAR1 or JA-Ile.

**Figure 1.**
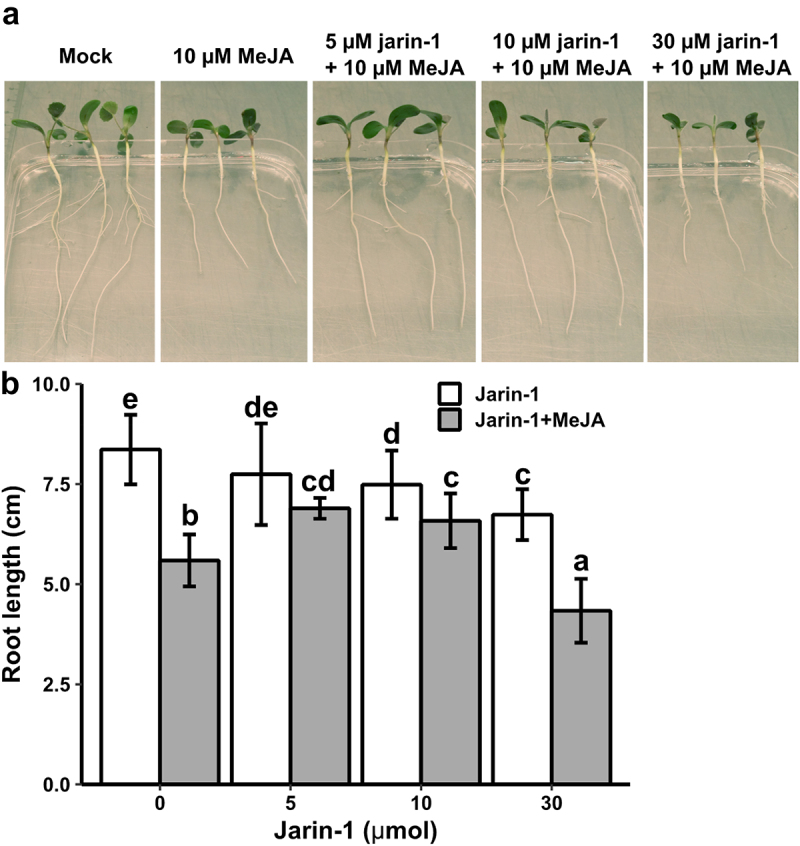
The effect of MeJA and jarin-1 on *M. truncatula* root growth. (a) Representative photographs of seedlings eight days after treatment with jarin-1 and MeJA. (b) Root length ±SD (*n*≥15). Four days after germination on agar plates in the dark, seedlings were placed into aluminium-wrapped 50 ml falcon tubes (without cover) filled with half-strength Hoagland’s solution supplemented or not with MeJA (10 µM) and/or jarin-1 (5, 10, 30 µM). There were eight treatments (tubes) in total, including mock, 10 µM MeJA, 5 µM jarin-1, 10 µM jarin-1, 30 µM jarin-1, 5 µM jarin-1 plus 10 µM MeJA, 10 µM jarin-1 plus 10 µM MeJA, 30 µM jarin-1 plus 10 µM MeJA. Seedlings were incubated for 8 days in a growth room at 24°C and 16 h light/8 h dark. Subsequently, seedlings were carefully removed from falcon tubes and photographed. Root lengths were determined using a ruler. Different letters indicate significant differences between treatments according to two-way ANOVA (*p*<0.05).

**Figure 2.**
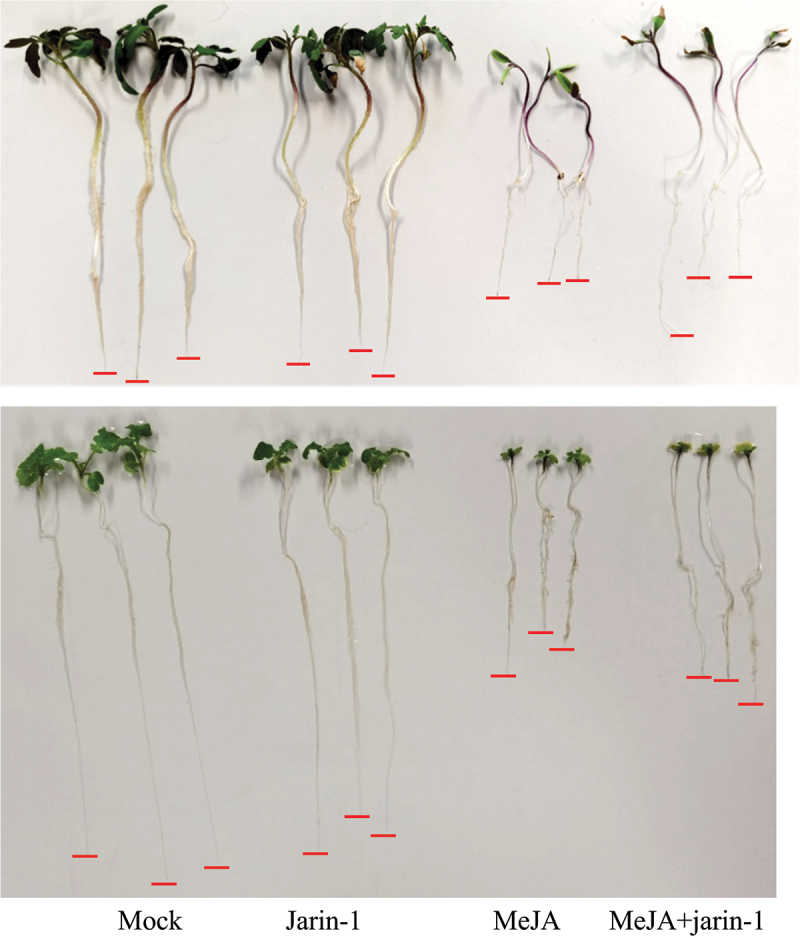
Effect of MeJA and jarin-1 on *S. lycopersicum* and *B. nigra* root growth. Representative photographs of seedlings (upper panel, *S. lycopersicum*; lower panel, *B. nigra*) 12 days after treatment with 10 µM jarin-1 and 10 µM MeJA. *S. lycopersicum* and *B. nigra* seedlings were placed in half-strength Hoagland solution. Four days after germination on agar plates in the dark, seedlings were placed into aluminium-wrapped 50 ml falcon tubes (without cover) filled with half-strength Hoagland’s solution supplemented or not with MeJA (10 µM) and/or jarin-1 (10 µM). There were four treatments (tubes) in total, including mock, 10 µM MeJA, 10 µM jarin-1, 10 µM jarin-1 plus 10 µM MeJA. Seedlings were incubated for 8 days in the growth room under conditions of 25°C (day)/21°C (night), 16‐h light: 8‐h dark. Subsequently, seedlings were carefully removed from falcon tubes and photographed.

**Figure 3.**
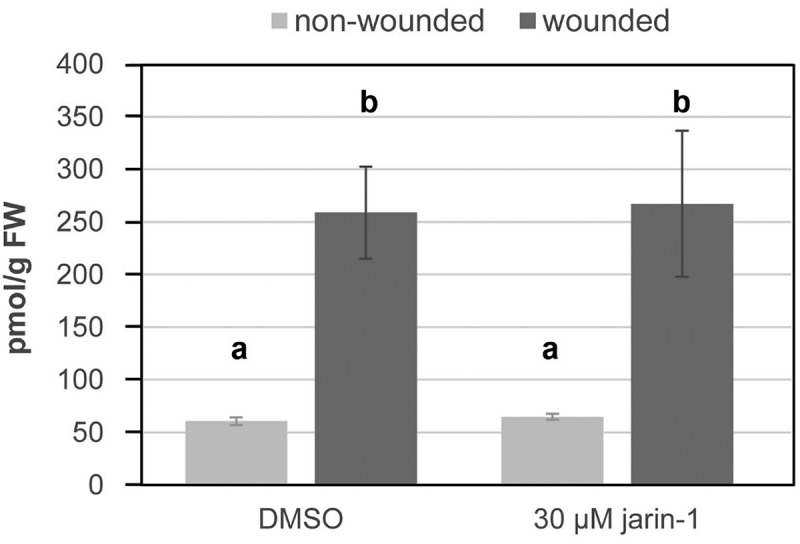
Application of jarin-1 did not affect wound-induced accumulation of JA-Ile in tomato leaf disks. Leaf disks of 6-week-old *S*. *lycopersicum* plants were placed on solutions containing DMSO (mock) or 30 µM jarin-1. After one hour, half of the disks were wounded using forceps and harvested one hour later. JA-Ile levels were determined according to Balcke *et al*.^16^. Bars represent mean ± SD (*n*=3). Different letters indicate statistically significant differences according to ANOVA with Tukey’s HSD test (*p*<0.01).

**Table 1.** Effect of jarin-1 on root length of *S. lycopersicum* and *B. nigra* seedlings 12 days after chemical treatment. Root lengths were determined using a ruler. Data are mean ± SD (n ≥ 7). DMSO (mock), dimethyl sulfoxide. Different letters indicate significant differences between treatments according to two-way ANOVA (p < 0.05).

Treatment	*S. lycopersicum* (cm)	*B. nigra* (cm)
DMSO	9.88 ± 1.83a	11.85 ± 1.72a
MeJA (10 µM)	3.76 ± 1.62b	3.81 ± 1.37b
Jarin-1 (10 µM)	9.90 ± 1.10a	12.97 ± 2.29a
MeJA+jarin-1 (10 µM each)	4.23 ± 2.02b	3.94 ± 1.48b

In conclusion, jarin-1 is a small-molecule inhibitor of jasmonate responses that is active in affecting the root growth inhibition by MeJA in *A. thaliana* and *M*. *truncatula*. However, in *S. lycopersicum* and *B. nigra*, we did not find this phenomenon under our experimental conditions. It still serves as an effective chemical tool in dissecting the complex jasmonate signaling networks to avoid the time-consuming genetic knockout for non-model plants. Its usability has, however, to be tested for other plant species.

## Data Availability

Data are available from the corresponding authors upon reasonable request.

## References

[1] Acosta IF, Farmer EE. Jasmonates. Arabidopsis Book. 2010;8:e0129. doi:10.1199/tab.0129. 22303255 PMC3244945

[2] Huang H, Liu B, Liu L, Song S. Jasmonate action in plant growth and development. J Exp Bot. 2017;68(6):1349–4. doi:10.1093/jxb/erw495. 28158849

[3] Wasternack C, Hause B. Jasmonates: biosynthesis, perception, signal transduction and action in plant stress response, growth and development. An update to the 2007 review in annals of botany. Ann Bot. 2013;111(6):1021–1058. doi:10.1093/aob/mct067. 23558912 PMC3662512

[4] Fonseca S, Chini A, Hamberg M, Adie B, Porzel A, Kramell R, Miersch O, Wasternack C, Solano R. (+)-7-iso-jasmonoyl-L-isoleucine is the endogenous bioactive jasmonate. Nat Chem Biol. 2009;5(5):344–350. doi:10.1038/nchembio.161. 19349968

[5] Yan J, Li S, Gu M, Yao R, Li Y, Chen J, Yang M, Tong J, Xiao L, Nan F, et al. Endogenous bioactive jasmonate is composed of a set of (+)-7-iso-JA-amino acid conjugates. Plant Physiol. 2016;172(4):2154–2164. doi:10.1104/pp.16.00906. 27756820 PMC5129707

[6] Guo Q, Major IT, Howe GA. Resolution of growth–defense conflict: mechanistic insights from jasmonate signalling. Curr Opin Plant Biol. 2018;44:72–81. doi:10.1016/j.pbi.2018.02.009. 29555489

[7] Karasov TL, Chae E, Herman JJ, Bergelson J. Mechanisms to mitigate the trade-off between growth and defense. Plant Cell. 2017;29(4):666–680. doi:10.1105/tpc.16.00931. 28320784 PMC5435432

[8] Züst T, Agrawal AA. Trade-offs between plant growth and defense against insect herbivory: an emerging mechanistic synthesis. Annu Rev Plant Biol. 2017;68(1):513–534. doi:10.1146/annurev-arplant-042916-040856. 28142282

[9] He Z, Webster S, He SY. Growth–defense trade-offs in plants. Curr Biol. 2022;32(12):R634–R639. doi:10.1016/j.cub.2022.04.070. 35728544

[10] Meesters C, Mönig T, Oeljeklaus J, Krahn D, Westfall CS, Hause B, Jez JM, Kaiser M, Kombrink E. A chemical inhibitor of jasmonate signaling targets JAR1 in *Arabidopsis thaliana*. Nat Chem Biol. 2014;10(10):830–836. doi:10.1038/nchembio.1591. 25129030

[11] Ishimaru Y, Hayashi K, Suzuki T, Fukaki H, Prusinska J, Meester C, Quareshy M, Egoshi S, Matsuura H, Takahashi K, et al. Jasmonic acid inhibits auxin-induced lateral rooting independently of the CORONATINE INSENSITIVE1 receptor. Plant Physiol. 2018;177(4):1704–1716. doi:10.1104/pp.18.00357. 29934297 PMC6084677

[12] Delgado LD, Zúñiga PE, Figueroa NE, Pastene E, Escobar-Sepúlveda HF, Figueroa PM, Garrido-Bigotes A, Figueroa CR. Application of a JA-Ile biosynthesis inhibitor to methyl jasmonate-treated strawberry fruit induces upregulation of specific MBW complex-related genes and accumulation of proanthocyanidins. Molecules. 2018;23(6):1433. doi:10.3390/molecules23061433. 29899259 PMC6100305

[13] Liu X, Cheng L, Li R, Cai Y, Wang X, Fu X, Dong X, Qi M, Jiang CZ, Xu T, et al. The HD-Zip transcription factor SlHB15A regulates abscission by modulating jasmonoyl-isoleucine biosynthesis. Plant Physiol. 2022;189(4):2396–2412. doi:10.1093/plphys/kiac212. 35522030 PMC9342995

[14] Munawar A, Xu Y, Abou El-Ela AS, Zhang Y, Zhong J, Mao Z, Chen X, Guo H, Zhang C, Sun Y, et al. Tissue-specific regulation of volatile emissions moves predators from flowers to attacked leaves. Curr Biol. 2023;33(11):2321–2329. doi:10.1016/j.cub.2023.04.074. 37224808

[15] Staswick PE, Sut W, Howell SH. Methyl jasmonate inhibition of root growth and induction of a leaf protein are decreased in an *Arabidopsis thaliana* mutant. Proc Natl Acad Sci. 1992;89(15):6837–6840. doi:10.1073/pnas.89.15.6837. 11607311 PMC49599

[16] Balcke GU, Handrick V, Bergau N, Fichtner M, Henning A, Stellmach H, Tissier A, Hause B, Frolov A. An UPLC-MS/MS method for highly sensitive high-throughput analysis of phytohormones in plant tissues. Plant Methods. 2012;8(1):47. doi:10.1186/1746-4811-8-47. 23173950 PMC3573895

